# Sophisticated Cloning, Fermentation, and Purification Technologies for an Enhanced Therapeutic Protein Production: A Review

**DOI:** 10.3389/fphar.2017.00419

**Published:** 2017-07-04

**Authors:** Sanjeev K. Gupta, Pratyoosh Shukla

**Affiliations:** ^1^Advanced Biotech Lab, Ipca Laboratories Ltd.,Mumbai, India; ^2^Enzyme Technology and Protein Bioinformatics Laboratory, Department of Microbiology, Maharshi Dayanand UniversityRohtak, India

**Keywords:** monoclonal antibody (mAb), cloning, therapeutic protein, drug development, quality by design (QbD), gene editing

## Abstract

The protein productions strategies are crucial towards the development of application based research and elucidating the novel purification strategies for industrial production. Currently, there are few innovative avenues are studies for cloning, upstream, and purification through efficient bioprocess development. Such strategies are beneficial for industries as well as proven to be vital for effectual therapeutic protein development. Though, these techniques are well documented, but, there is scope of addition to current knowledge with novel and new approaches and it will pave new avenues in production of recombinant microbial and non-microbial proteins including secondary metabolites. In this review, we have focussed on the recent development in clone selection, various modern fermentation and purification technologies and future directions in these emerging areas. Moreover, we have also highlighted notable perspectives and challenges involved in the bioengineering of such proteins, including quality by design, gene editing and pioneering ideas. The biopharmaceutical industries continue to shift towards more flexible, automated platforms and economical product development, which in turn can help in developing the cost effective processes and affordable drug development for a large community.

## Introduction

Commercial production of recombinant therapeutic proteins including monoclonal antibodies (mAbs) is one of the therapeutic areas that have undergone remarkable improvements with the implementation of various novel technologies over the last decade or so. To minimize the manufacturing cost and timeline, platform technologies have been developed for the similar categories of drug and the regulatory requirements, which includes an engineered production host, cell line screening and selection devices, media and feed selection, advancement in upstream and downstream processes and process intensification by implementing continuous manufacturing. In addition, a revolution in the use of single use devices has improved and simplifies the production processes significantly, which offers a cost-effective product development for small-scale to mid-scale production processes (Chon and Zarbis-Papastoitsis, 2011).

The bacterial host *Escherichia coli* is the most popular expression system used for the production of a quite good number of recombinant proteins (Akesson et al., 2001; Jana and Deb, 2005; Shrivastava et al., 2013). Several recombinant proteins, including biopharmaceutical products (**Table 1**) have been developed and launched using *E. coli* as an expression host (Olempska-Beer et al., 2006; Huang et al., 2012; Gupta and Shukla, 2015; Mane and Tale, 2015).

**Table 1:** Recombinant Proteins produced by different hosts (Olempska-Beer et al., 2006; Fakruddin et al., 2013; Gupta and Shukla, 2015; Mane and Tale, 2015 and www.fda.gov).

Sr. No.	Product category	Product name
1	Industrial enzymes	Amylases, lipase, phytase, laccase, chymosin, glucose oxidase, pullulanase, enterokinase, invertase, cellulase, xylanase, etc.
2	Therapeutic proteins	Insulin, G-CSF, GM-CSF, insulin glargine, insulin lispro, HSA, Fab and ScFv fragments, FSH, EPO, TPA, monoclonal antibodies, recombinant vaccines, interferons, etc.
3	Therapeutic enzymes	Streptokinase, urokinase, trypsin, glutaminase, B-lactamase, L-asparaginase, glucosidase, collagenase, uricase, etc.

*Escherichia coli* has been proven as a best expression host for the production of non-glycosylated proteins as it offers various advantages over yeast and other expression systems due to its well-understood genetics, cell biology, easy handling and simple upstream process (USP) which allows production of cost-effective large quantity of recombinant proteins. Recently, several recombinant therapeutic proteins and industrial enzymes are produced using *E. coli* expression system (**Table 1**) (Fakruddin et al., 2013; Spadiut et al., 2014; Mane and Tale, 2015). *E. coli*, produces recombinant proteins, mainly three different forms such as inclusion body, the secretary as well as soluble forms (Fahnert et al., 2004; Zerbs et al., 2014). In addition, various novel recombinant proteins are still being produced in this system today (Wells and Robinson, 2016).

The second and third most favorable microbial systems after *E. coli* for the production of recombinant proteins is a eukaryotic microorganism *Saccharomyces cerevisiae and Pichia pastoris*, respectively. It has the ability to produce therapeutic proteins with post-translational modifications closer to human. *Saccharomyces cerevisiae and P. pastoris* is being used for several decades for bakery and brewing industries; however, it has also been used for the production of many recombinant therapeutic proteins (**Tables 1**, **2**) at industrial scale. Both the hosts are capable of producing recombinant proteins with proper folding and post-translational modification (Dalton and Barton, 2014) closure to human, therefore these systems are considered as better than prokaryotes where post-translational modification of the protein is required.

**Table 2:** Novel recombinant proteins produced by different expression systems, 2011–2015 [Wells and Robinson, 2016 and New Drugs at FDA: CDER’s New Molecular Entities and New Therapeutic Biological Products (http://www.fda.gov/Drugs/DevelopmentApprovalProcess/DrugInnovation/), 2016].

Sr. No.	Generic name	Brand name	Year	Production host
1	Teduglutide	Gattex	2012	
2	Tbo-filgrastim	Neutroval	2012	*E. coli* (Bacteria)
3	Glucarpidase	Voraxaze	2012	
4	Metreleptin	Myalept	2014	
5	Parathyroid hormone	Natpara	2015	
1	Ocriplasmin	Jetrea	2012	*P. pastoris* (Yeast)
2	Albiglutide	Tanzeum	2014	*S. cerevisiae* (Yeast)
1	Ipilimumab	Yervoy	2011	
2	Aflibercept	Eylea	2011	CHO (Mammalian)
3	Brentuximab vedotin	Adcetris	2011	
4	Ziv-aflibercept	Zaltrap	2012	
5	Pertuzumab	Perjeta	2012	
6	Obinutuzumab	Gazyva	2013	
7	Ado-trastuzumab emtansine	Kadcyla	2013	
8	Peginterferon beta-1a	Plegridy	2014	
9	Vedolizumab	Entyvio	2014	
10	Siltuximab	Sylvant	2014	
11	Blinatumomab	Blincyto	2014	
12	Daratumumab	Darzalex	2015	
13	Mepolizumab	Nucala	2015	
14	Asfotase alfa	Strensiq	2015	
15	Idarucizumab	Praxbind	2015	
16	Evolocumab	Repatha	2015	
17	Alirocumab	Praluent	2015	
18	Secukinumab	Cosentyx	2015	

However, the fourth most popular host mammalian system which is used for the production of around 70% recombinant proteins so far developed using the host Chinese hamster Ovary (CHO) cells (Jayapal et al., 2007). In addition, other mammalian cells such as mouse myeloma cells (Sp2/0 and NS0) and Baby hamster kidney (BHK-21) cells are also being used for the production of couple of recombinant therapeutic proteins. The major advantage of mammalian cells over above described system is that this system produces recombinant proteins with the most closure to human glycosylation and other post-translational modifications with the highest reliability (Dalton and Barton, 2014). A therapeutic enzyme, Activase^®^ was the first FDA approved recombinant protein developed in mammalian CHO cells in 1987 (Jayapal et al., 2007), since then over 100 recombinant proteins so far has been made using the mammalian system. Since 2011, FDA has approved 48 novel therapeutic proteins (**Table 2**) out of which 29 are recombinant mAbs produced either by CHO or mouse myeloma cells. The total sale of one of the blockbuster mAbs Adalimumab (Humira) has reached to $12.5 billion in 2014–2015 (Morrison, 2015).

Other expression systems used for the production of recombinant therapeutic proteins include insect derived cell lines Sf9 and Sf21 from *Spodoptera frugiperda*, tobacco plant (*Nicotiana tabacum*), transgenic animals (*Mus musculus*, *Bos taurus*), and other fungi (*Aspergillus niger*), however, the focus of this review is on *E. coli*, *S. cerevisiae*, *P. pastoris*, and mammalian CHO cells used frequently for therapeutic product development (Wells and Robinson, 2016).

**FIGURE 1:**
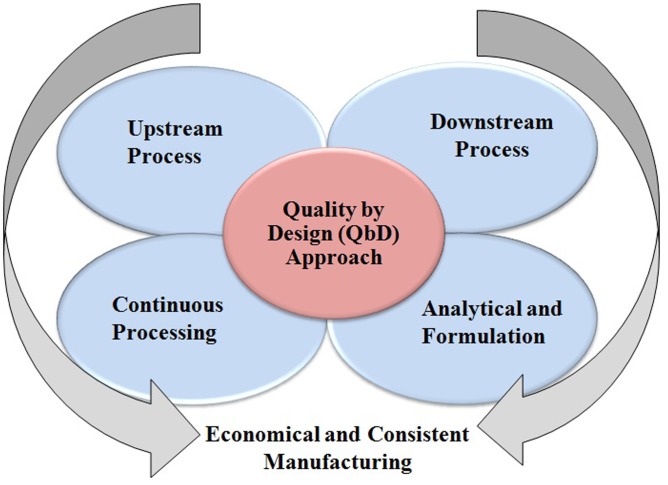
Implementation of QbD approach for the production of therapeutic drugs.

Among the therapeutic proteins produced by mammalian system, the mAbs are used globally in diverse applications such as bio-therapeutic as well as diagnostic purposes (Jain and Kumar, 2008; Shukla and Thömmes, 2010; Elvin et al., 2013). However, USP at low productivity is more costly than downstream process. Furthermore, the innovative technologies are implemented in purification processes to address the “downstream bottlenecks” which allows easy handling of high titer volume in a production process. Innovation in manufacturing processes helps in driving the improved production cost, flexibility, and product quality which is ultimately beneficial to the end-users. These innovative technologies include: (1) Use of single-use systems in upstream and downstream processes, (2) Continuous manufacturing/process intensification to reduce the manufacturing footprint and economical production, (3) Alternative purification processes such as membrane chromatography for an efficient purification, and (4) Implementation of Quality by Design (QbD) approach for a successful and economical process development (**Figure 1**) (Strube et al., 2014).

This review summarizes the innovative approaches in cell line development, upstream and downstream processes including use of a single-use holistic process and facility for efficient bacterial, yeast and mammalian based recombinant product development. We also discussed that how the implementation of the QbD approach in both the processes ensures consistency in the process as well as product quality. The use of single use system and continuous manufacturing enables economical manufacturing to support the increasing demand of affordable biologics also described.

## Expression Hosts for Protein Production

Among the expression systems used by various industries, the bacterial *E. coli*, yeast *S. cerevisiae* and Mammalian CHO cell line are mostly used for the production of various recombinant therapeutic proteins. These proteins are successfully commercially launched worldwide. A brief description of advances in these systems is described below.

### *E. coli* (Bacterial)

The *E. coli* system is relatively simple due to ease of handling, basic nutritional requirement, easy genetic manipulation, including gene cloning and cell engineering, signal transduction, easy fermentation process development (Razzell and Khorana, 1958; Pardee et al., 1959; Yanofsky and Lennox, 1959). However, the purification process is relatively cumbersome due to lack of post-translation modification machinery which leads to less final recovery compared to other two expression systems.

The other disadvantage of bacterial system is the presence of endotoxins which has the potential safety concern as the patients medicated with *E. coli*-produced recombinant proteins may show the immune response to the drug administered to the patients, so improved removal of these contaminants through purification process will lead to increased safety of bacterial derived therapeutics (Poltorak et al., 1998; Hoshino et al., 1999; Mamat et al., 2015).

For proper folding of the expressed protein, the gene encoding disulfide isomerase has been stably integrated in the genome of *E. coli* which has demonstrated improved cytoplasmic protein folding and solubility (Lobstein et al., 2012). For example the modified strain SHuffle (Lobstein et al., 2012) has recently been developed for the production of full length, effector-binding IgGs (1–25 mg L^-1^) without *in vitro* refolding post expression (Robinson et al., 2015). In addition, for efficient secretion of recombinant proteins produced in *E. coli* many periplasmic such as DsbAss, MalEss, and OmpAss and extracellular signal sequences are incorporated along with the desired gene, which has successfully led to protein secretion in periplasmic as well extracellular space of the *E. coli* cells, which ultimately led to soluble protein expression in *E. coli* (Schierle et al., 2003; Sockolosky and Szoka, 2013; Schlegel et al., 2013).

The *E. coli* cells are also engineered for glycosylation of recombinant protein produced using engineered cells. Szymanski et al. (1999) identified general *N*-linked protein glycosylation in the bacterium *Campylobacter jejuni* (Szymanski et al., 2003), these strains have the potential to produce *N*-glycosylating several recombinant proteins (Wacker et al., 2002; Fisher et al., 2011).

### *S. cerevisiae* (Yeast)

The *S. cerevisiae* yeast expression system is frequently used for recombinant protein production due to their rapid growth in protein-free media, the presence of post-translational modifications, machinery, and ability to secrete the product extracellularly (Tyo et al., 2014; Tang et al., 2015).

However, overexpression of recombinant protein may cause intracellular accumulation and reduced yields (Idiris et al., 2010; Tyo et al., 2014). With regard to post-translational modifications that occur within the cells often lead to the production of undesired hypermannosylation proteins which may lead to faster blood clearance when administered to the human body (Wildt and Gerngross, 2005). To overcome with such issue, the gene encoding mannosyltransferase has been knocked out by Nasab et al. (2013) in which they generated an ALG3/ALG11 double knockout *S. cerevisiae* which prevented hypermannosylation of the expressed protein.

### *P. pastoris* (Yeast)

**FIGURE 2:**
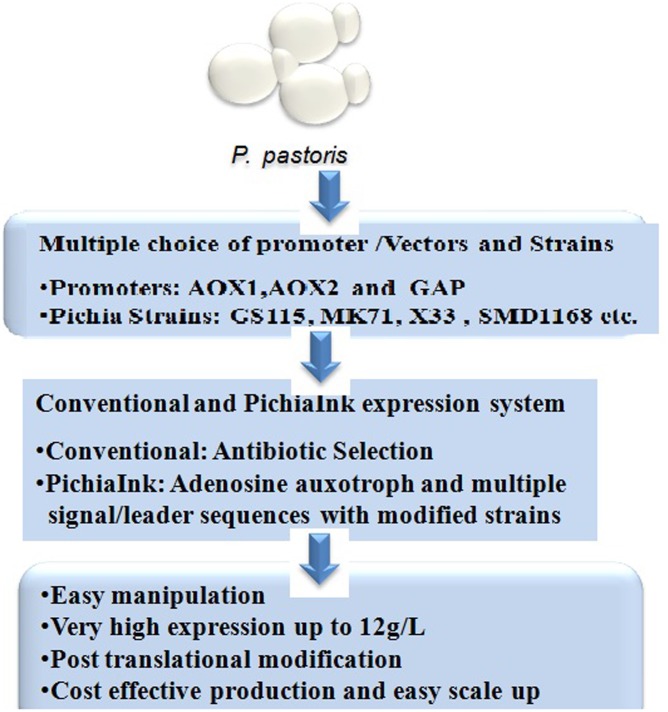
*P. pastoris* expression system and its advantages.

The methylotrophic yeast *P. pastoris* is an established industrial host for the recombinant protein expression largely used for the commercial production of various enzymes and therapeutic proteins (Kurtzman, 2009). It was introduced for over four decades by Phillips Petroleum for the commercial production of single cell protein (SCP) for the use of animal feed additive. Later in the 1980s, *P. pastoris* was developed as a recombinant protein expression host using the strong and tightly regulated alcohol oxidase (AOX1) promoter (Cregg et al., 1985). This system is potentially used for the production of recombinant proteins in both secretary extracellular as well as intracellular manner. *P. pastoris* grows with very high cell density in fermentation culture thus produces large quantity of desired proteins (Ahmad et al., 2014). The strong and tightly regulated promoters used for protein expression is one of the major elements enables high protein expression. In the1990s, *P. pastoris* was used first time for the industrial production of the plant-derived enzyme hydroxynitrile lyase at over 20 g/L of culture supernatant (Hasslacher et al., 1997). *Pichia* expression system offers several advantages over other expression systems (**Figure 2**), these includes: (1) Suitable host for high expression with several post-translational modification, (2) Grows easily with very high cell density in the defined medium, (3) Easy scale up in large scale fermentation, and (4) Cost effective process and product development, relatively cheaper than mammalian process^1^.

### CHO Cells (Mammalian)

Mammalian cells are mostly used for the production of recombinant therapeutic proteins as this allows to generate more humanlike protein. Mammalian cells demands richer medium and feed for their growth, stringent growth conditions and sustained growth period than microorganisms (Birch and Racher, 2006; Huang et al., 2010). The gene introduction and clone development in mammalian system is more tedious than microbial system. Several transfection methods have been developed and attempted for a successful gene introduction and pool generation with high transfection efficiency. However, recently developed, ligase independent cloning and screening techniques allow for more efficient cloning and expression of desired proteins with superior characteristics (Green and Sambrook, 2012). However, the cell line still requires several modifications to achieve high expression and good product quality (Wells and Robinson, 2016).

## Manufacturing Process Development

Implementation of innovative platform technology is possible in research and development followed by in manufacturing. These platform technologies for recombinant protein production are used mainly for the development and optimization of upstream and downstream processes. These technologies are adopted in cell line development, clone screening and selection, medium and feed optimization, process optimization and cell clarification methods on upstream side, (Birch and Racher, 2006; Shukla and Thömmes, 2010) and the purification optimization of individual operation on the downstream side (Liu et al., 2010).

The fermentation process of microbial system requires analysis of spent medium which gives an appropriate idea about consumption and accumulation profile of components present in the culture supernatant. Furthermore, the medium feed and bioreactor process are optimized based on the understanding developed from spent medium analysis (Challener, 2015). Various carbon sources are used in *E. coli* and yeast culture as the main medium components in the bioproduction, which may affect the cell metabolism, protein production, and protein quality, significantly (Seyis and Aksoz, 2005).

Clone screening, medium and feed screening, and process parameters remains challenging at an early stage of development. However, with the advent of high throughput screening devices such as Mini-bioreactor it has now become easy to perform all screening work including process optimization at very small scale. Use of such advanced microbioreactor system allows a potential time and cost savings involved in microbial fermentation process development for large scale recombinant protein production (Gupta and Shukla, 2015).

Very recently, another small microbioreactor has been introduced by M2p-labs that is called BioLector^®^. This platform contains 48 microbioreactor which is used to perform high-throughput fermentations together with online monitoring of the process parameters such as biomass, pH, DO, florescence, etc. The microtiter plate is used in the BioLector^®^ micro-bioreactor which operates with non-invasive, optical sensors. In addition, the Biolector controls the shaking speed, the temperature as well as the humidity. This platform is ideal for both aerobic and anaerobic cultures. To date, this system is used for cultivation and process development of various microorganisms such as *E. coli*, *B. subtilis*, *Lactobacillus*, *P. pastoris*, *H. polymorpha*, *S. cerevisiae*, *Streptomyces*, *Penicillium*, *Tetrahymena*, *Sf9/SF21*, *N. tabacum*, etc.

The Biolector system is used for a broad range of applications viz. cell line and strain screening, media and feed screening, Fermentation process parameter optimization, synthetic and systems biology, anaerobic and microaerophilic fermentation, design of experiments, growth, characterization, protein kinetics study, high-throughput protein expression, Enzyme and cell activity test, proteomics studies and quality control etc.^2^

The high-throughput screening devices used at early development stage have greatly helped in reducing the intense time pressure in the manufacturing process (Amanullah et al., 2010; Bhambure et al., 2011) as these devices allows running a great number of clones and process screening experiments at small scale with the use of bare minimum consumable items. For example, use of HTP device Advanced micro-bioreactor (AMBR) has been potentially used for the cell line selection, medium feed screening and process optimization at very small scale (10–15 mL).

Furthermore, for an efficient development of manufacturing processes, the concept of QbD amalgamated with high throughput methods or DoE (Michels et al., 2012; Pathak et al., 2014) are highly recommended to be applied in both upstream, downstream as well analytical processes (Rathore, 2009; del Val et al., 2010; Jiang et al., 2010; Martin-Moe et al., 2011). The QbD method is implemented for the development of more robust and proficient production processes for recombinant proteins including mAbs with augmented clinical efficacy (del Val et al., 2010). Horvath et al. (2010) described a QbD-based USP optimization (**Figure 1**). Harms et al. (2008) and Abu-Absi et al. (2010) studied and published the mapping design space for upstream cell culture and fermentation processes. Jiang et al. (2010) published important findings on the application of QbD principles for hydrophobic interaction chromatography (HIC) and Pathak et al. (2014) as well as Michels et al. (2012) published article on QbD-based analytical method development for mAb aggregates and size heterogeneity analysis.

## Clone and Upstream Development

Upstream process development and process tweaking includes process development and optimization. USP includes diverse parts, such as cell line engineering and stable cell line development, high expressive clone selection, media and feed screening, process development and scale up from small scale to manufacturing scale (Li et al., 2010; Rita Costa et al., 2010; Butler and Meneses-Acosta, 2012; Zhu, 2012; Yang and Kiu, 2013). In addition, the bio-reactor configuration and its design, cell clarification method, process control and the analytics can be considered as part of the optimization process (Rita Costa et al., 2010; Butler and Meneses-Acosta, 2012; Yang and Kiu, 2013). A thorough process optimization approach leading to the generation of a high product titer, high yield, and desired product quality (Kelley, 2007; Li et al., 2010; Rita Costa et al., 2010).

### Cell Line Engineering and Clone Selection

#### *E. coli* Clone Development

Among the three expression system, the *E. coli* system is relatively simple due to ease of handling, basic nutritional requirement, easy genetic manipulation, including gene cloning and cell engineering, signal transduction, easy fermentation process development (Razzell and Khorana, 1958; Pardee et al., 1959; Yanofsky and Lennox, 1959). However, the purification process is relatively cumbersome due to lack of post-translation modification machinery which leads to less final recovery compared to other two expression systems. The desired gene can be easily inserted into a bacterial plasmid and amplified in the host via bacterial transformation followed by antibiotic selection. A new plasmid may take as less as few days for the gene cloning and protein expression studies (Sambrook et al., 1989). Now-a-days, a wide variety of bacterial expression plasmids and modified *E. coli* hosts are available from various commercial sources (e.g., Novagen) for the gene cloning and protein expression studies followed by industrial protein production (Huang et al., 2012).

Major differences among the expression plasmids includes the availability of different antibiotic selection markers, gene induction method, with or without signal sequence and modified expression cell line for example protease deficient *E. coli* BL21DE3 cells and may have specialized protein folding machinery such as co-expression with the chaperons for improved protein folding thus efficient production (Huang et al., 2012; Lobstein et al., 2012). The bacterial expression system is also used for the production of antibody fragments and its derivatives (e.g., ScFv, Fab, etc.) for the production at commercial scale and therapeutic human use (Gupta and Shukla, 2016a). Despite various advantages, the major disadvantage of the *E. coli* system is lack of post-translational machinery which leads to cumbersome expression and purification development (Mamat et al., 2015).

#### *S. cerevisiae* Clone Development

For clone development in yeast *S. cerevisiae*, single-copy and multi-copy vectors have been established for decades now for potential use as an expression plasmid to produce recombinant proteins. When these vectors are transformed in yeast unlike the *E. coli* system the desired genes are stably integrated to the host genome and provide stable cell line for commercial large scale protein production. Integration of high gene copies allows greater overall protein production in the production reactors (Boder and Wittrup, 1997; Cregg et al., 2010). To increase the gene expression, the rationally designed or fully synthetic promoters have been successfully attempted in *S. cerevisiae* (Curran et al., 2014).

Curran et al. (2014) have developed a stronger promoter by lowering its nucleosome affinity, and were able to achieve up to 16-fold increased transcriptional activity of a modified yeast CYC1 promoter compared to wild type CYC1.

#### *P. pastoris* Clone Development

The *P. pastoris* system has flexibility of selecting an appropriate vector and compatible host for high and economical expression of recombinant proteins which are the most important and prerequisite for successful product development. Similar to *S. cerevisiae*, in *P. pastoris* also single to multiple genes can be integrated to the genome for high expression of desired protein. The use of a strong and tightly regulated alcohol inducible Promoter (*AOX*) enables overexpression of recombinant protein. In *Pichia*, two genes *AOX1* and *AOX2* codes for alcohol oxidase mainly accounts for alcohol oxidation in the cells. Expression of *AOX1* is induced with methanol to extremely high level over 30% of the total soluble proteins of the cells. The *AOX1* gene has been incorporated in the *Pichia* expression vector to drive high protein expression of desired gene (Ellis et al., 1985; Tschopp et al., 1987; Koutz et al., 1989). Whereas the second gene *AOX2* is about 97% homologous to *AOX1 gene*, used for isolation of MutS strain as cells grows much slower in methanol than with *AOX1* (Cregg et al., 1989; Koutz et al., 1989). In presence of carbon source such as glucose, glycerol, or ethanol, the promoter *AOX1* is strongly repressed (Inan and Meagher, 2001). Furthermore, the promoter is de-repressed upon depletion of above carbon source, and fully induced only when methanol is added for induction and protein production.

Multiple vectors (pPIC9, pPICZα-A, B, and C, etc.) and strains (GS115, KM71, X33, etc.) have been developed and commercially available (Thermo Scientific/Life Tech, United States) for ease of selection and cloning of desired gene for high level protein expression (**Figure 2**).

In addition to inducible promoter, *P. pastoris* vectors are available with the glyceraldehyde-3-phosphate promoter (PGAP) constitutive promoter which gives almost similar expression level in presence of glucose that of methanol inducible *AOX* promoter (Waterham et al.1997).

Recently, a novel platform PichiapInk expression system has been introduced by Thermo Scientific which is more advantageous than conventional existing *Pichia* expression system (**Figure 2**). The clones are selected using ADE3 complementation (complementation of Adenine auxotroph) rather than antibiotic resistance unlike conventional *Pichia* expression system. A very high protein expression up to 12 g/L has been achieved using PichiapInk system^3^. Following are the main features of PichiapInk system:

(1) Both low and high copy (LC and HC) number plasmids are available which enables optimization of expression of toxic proteins.
(2) Multiple up to eight secretion and leader sequences are available for protein secretion.
(3) Four strains are available for transformation and protein expression optimization.
(4) Three protease-deficient host strains are available.
(5) Easy scale up from small to large scale fermentation.

#### CHO Clone Development

A cell line for any biopharmaceutical manufacturing is a starting material. The cell line engineering includes, host and vector selection, metabolic engineering by gene modulation and stable commercially viable clone selection. The major step involved in cell line development, is a selection of an expression host, appropriate compatible expression vectors, transfection as well as cell line selection. Various high throughput devices such as CLonePix (Thermo) and FACS (BD and Beckman) are now-a-days potentially used for the cell line development and its screening. The selection of an expression platform is determined by its capability to give high productivity with desired product quality (Li et al., 2010; Rita Costa et al., 2010; De Jesus and Wurm, 2011). The CHO cells are mostly used as a host for the production of recombinant proteins, including mAbs and fusion proteins (Jayapal et al., 2007; Kelley, 2009; Li et al., 2010; Rita Costa et al., 2010; De Jesus and Wurm, 2011; Gupta and Shukla, 2015, 2016a). Recombinant interferons and tissue-type plasminogen activator (tPA) were the first proteins produced by CHO cells (De Jesus and Wurm, 2011). **Table 3** summarizes the conventional and innovative technologies and their advantages for an affordable drug development.

**Table 3:** Summary of conventional and innovative approaches in manufacturing of recombinant products (del Val et al., 2010; Liu et al., 2010; Rita Costa et al., 2010; Shukla and Thommes, 2010; Bhambure et al., 2011; Angarita et al., 2015; Klutz et al., 2015; Zhao et al., 2015; Gupta and Shukla, 2016b).

Sr. No.	Process/step	Conventional approach	Innovative approach	Advantages
1.	Cell Line Development	1. Use of Amplification marker(i.e., DHFR) 2. Random integration in CHO genome 3. Manual cloning and screening	1. Site specific integration at transcriptionally active site using gene editing. 2. Site specific integration 3. Use of HTP devices such as CLonePix, FACS, Biolector and AMBR systems	1. Development of stable clones 2. Rapid screening of clones 3. Selection of good quality clones 4. Time and cost saving
2.	Upstream process development	1. Shake flask study 2. Bioreactor study 3. Scale up in stainless steel bioreactors 4. Perfusion process	1. Use of AMBR and Biolector for media/feed and process screening 2. Use of multiple small scale bioreactors 3. Use of single-use bioreactors and components 4. Perfusion process using new devices	1. Rapid screening and process optimization at small scale 2. DOE can be implemented easily in AMBR/Biolector for a robust process development 3. Cost and time saving 4. Minimizes chance of contamination 5. Easy validation
3.	Downstream process development	1. Use of AKTA system 2. Centrifugation for harvest clarification 3. Resins and column	1. Use of AKTA_Avant with inbuilt DOE system 2. Single use depth filtration and flocculant 3. In-line dilution and In-line conditioning systems 4. Use of BioSMB and PCC systems for continuous downstream 5. Use of single use technology and component	1. Easy resin and process selection 2. Robust DSP process 3. Efficient harvest clarification with depth filters 4. Reduced cost, reduced foot print and labor cost 5. Economical process development
4.	Upstream and Downstream process	1. Separate operation	1. Integration of both the processes 2. Continuous processing	1. Reduced foot print in the manufacturing 2. Economical process development 3. Affordable drug development

The high protein expression with the desired product quality in terms of post-translational modification and genetic stability are the major criteria for a clone selection after extensive screening. In addition, other characteristics such as cell growth pattern, stable and consistent production, cultivation as suspension culture in serum free medium, scalability in the bioreactor and adaptive performances are also considered while clone development and its selection (Li et al., 2010; De Jesus and Wurm, 2011). Furthermore, several analytical methods are employed while clone and process selection to ensure selection of the desired clone giving improved expression and good product quality such as glycosylation pattern (Durocher and Butler, 2009; Li et al., 2010; Zhu, 2012).

**FIGURE 3:**
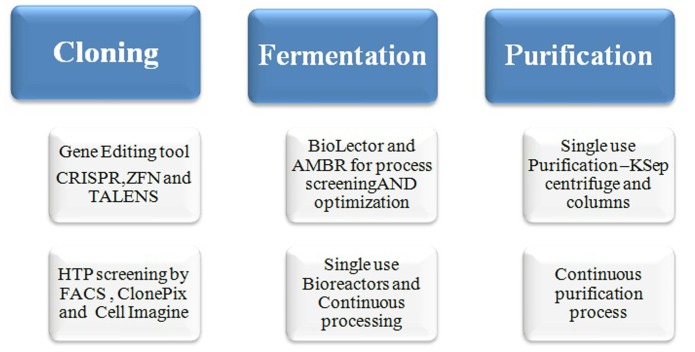
Modern clone and process development approaches.

Some of the innovative approaches such as metabolic engineering using the gene editing tools for knocking-in and knocking-out of a particular gene at specific loci of the host cells are now-a-days practiced for an efficient clone and product development (Gupta and Shukla, 2016b). The gene editing tools, CRISPR/Cas9, TALENs, and ZFNs are most commonly used for the host engineering (**Figure 3**), which may result developing a stable and high producer clone with consistent product quality (Gupta and Shukla, 2016b). Furthermore, glycoengineering is another approach adopted for the production of desired glycoform and good quality product for improved potency. The metabolic engineering of the cells allows controlled or less accumulation of waste product such as ammonium and lactic acid (Mori et al., 2004). According to the report of “Analysis and Global Forecast 2019” the market for global Cell line development is expected to reach to $3.94 billion by 2019 which has been $2.2 billion in 2014. Genome editing market size is subdivided into CRISPR/Cas9, Zinc Finger Nucleases (ZFNs), Meganucleases, and TALENs. The use of endonucleases for the manipulation of cells is advanced and recent approach which allows a precise and site-specific editing of the host genome. These tools are extremely used now-a-days by the Biopharma Industries for cell line engineering and bioproduction. It can be potentially employed to generate better cell factories for the bio-production recombinant proteins. The manipulations that can be done with CRISPR have been done earlier by using other gene editing tools such as transcription activator-like effector nucleases (TALENs) and ZFNs (**Figure 3**). Many researchers have found CRISPR as a less tedious and more efficient tool as compared to above described two other gene editing tools (Hou et al., 2015; Gupta and Shukla, 2016b; Savić and Schwank, 2016).

Commonly used production hosts such as yeast, *E. coli* and mammalian CHO cells are being used for the production biopharmaceuticals, however, the CRISPR/Cas9 tool is frequently used for the yeast and CHO cell engineering. However, a limited research has been conducted so far on the application of CRISPR in bacterial system. Two basic strategies are followed for the gene editing using CRISPR platform, Gene Knock-out (KO) and Gene Knock-In (KI). In a KO, the gene is not transcribed at all, while in Knock-in, part of any gene inserted at the site-specific which is transcribed in the host (Hou et al., 2015; Gupta and Shukla, 2016b; Savić and Schwank, 2016).

Metabolic engineering of the *E. coli* cells require chromosomal integration of single or multiple genes, however, the integration of large DNA into *E. coli* remains challenging. With the advent of CRISPR/Cas9 it is now possible to integrate large DNA in *E. coli*. Very recently, Chung et al. (2017) demonstrated that CRISPR/Cas9 system can be used for the chromosomal integration of large DNA into *E. coli.* The CRISPR/Cas9 driven homologous recombination allowed replacement of *lac*Z gene in the MG1655 strain with efficiency up to 99%, and also enabled high fidelity, scarless integration of 2.4, 3.9, 5.4, and 7.0 kb DNA with the efficiencies of 91, 92, 71, and 61%, respectively. It is also reported that the CRISPR/Cas9 approach was also able to integrate functional genes in diverse *E. coli* strains, including BL21 (DE3) (Chung et al., 2017).

Metabolic pathway engineering is one of the most recent approaches implemented for improved production of heterologous proteins in yeasts. This is also implemented for efficient production of biofuels (Krivoruchko and Nielsen, 2014). The gene editing tools such as CRISPR/Cas9 complex can be potentially used for yeast engineering for site specific gene integration or knock-out of certain unwanted genes in an improved protein production.

Recent data suggest that, most of the complex therapeutic proteins including mAbs are produced from the popular host CHO cells due to its ability to produce correctly folded and glycosylated proteins. In addition, certain genes such as LDH (lactate dehydrogenase) and FUT8 (fucosyltransferase 8) have been knocked-out in CHO cells for the improvement of lactate metabolism as well as product quality, respectively (Hou et al., 2015; Gupta and Shukla, 2016b; Savić and Schwank, 2016). With the advent of gene editing tools CRISPR/Cas9 technology, it has become easy to modify the CHO cell line for deletion or gene integration at specific loci in the genome (Grav et al., 2015).

Several conventional methods such as codon optimization, gene amplification using a different selection marker (i.e., DHFR/MTX), single cell dilution cloning and screening have been practiced much for a recombinant cell line development (Zhu, 2012).

Recently, the cell line is engineered by gene knockout for gene encoding FUT8 enzyme which have shown improved antibody-dependent cell-mediated cytotoxicity (ADCC) effector functions for antibodies produced in those lines (Yamane-Ohnuki et al., 2004). With the advent of gene editing tools CRISPR/Cas9 technology, it has become easy to modify the CHO cell line for deletion or gene integration at specific loci in the genome (Grav et al., 2015). Another gene editing tool ZFNs has been used for the downregulation of apoptotic genes (e.g., BAX/BAK) for the improved protein production in mammalian cells (Lim et al., 2006; Cost et al., 2010).

Subsequently, the best clone is selected based on the process feasibility, upstream suitability, its behavior in the bioreactor and product quality attributes as any variation in the selected clone during the clinical development or manufacturing represent a most important process change which further requires additional comparability study. The final clone selected for the bioproduction should fulfills the desired product quality, titer, specific productivity, process feasibility, expected charge variant and glycosylation profile, no or less aggregate formation and clone stability among others (Lee et al., 2009; Li et al., 2010; Rita Costa et al., 2010; Noh et al., 2013). In addition, metabolic behavior in terms of lactic acid and ammonium accumulation profiles are to be considered for a better process development and product quality. Ultimately, overall performance in a reactor decides which clone is to be considered for the bioproduction and commercialization since the product quality, titer and metabolic behavior of the clone is sturdily depends on the upstream cell culture process developed in a bioreactor (Li et al., 2010).

### Upstream Process Development (Fermentation)

The next important step in bioproduction after clone selection is cell culture USP development. Various new tools and techniques are evolving recently for a better process control and cell culture process optimization. “Use of the single use bioreactor system with well controlled automation is a new trend now-a-days in the Biopharma companies.” The single use system requires lower capital investment and operational cost, improved production campaign, process reproduction and better flexibility as compared to the conventional stainless steel system. These single use bioreactors and other accessories are available from 50 to 2000 L scale (Langer and Rader, 2014). Disposable bioreactors are now-a-days available in different forms such as stirred-tank, wave reactor, orbital shaken, etc. (Whitford, 2010; Shukla and Gottschalk, 2013). They reduce the chance of contamination significantly. A new single use orbital shaken bioreactor up to 2500 L scale has been developed which has cylindrical or square shaped vessel (De Jesus and Wurm, 2011). The concept and fluid design model and vessel of this bioreactor were first published by Reclari et al. (2014).

Other single-use bioreactors have been designed based on a bag concept which is used in both research as well as Biomanufacturing of recombinant products. However, the mixing and gassing strategies vary in each of them, therefore, still a lot of work is being done for optimizing single-use bioreactor systems (Shukla and Gottschalk, 2013; Minow et al., 2014). In addition to these advantages, the single-use systems also have few limitations in terms of product safety due to the risk of leachable and extractable from the disposable plastics (Whitford, 2010; Löffelholz et al., 2013; Shukla and Gottschalk, 2013). The plastic materials used in production bag can also bind with media components and leads to decreased processing performance (Shukla and Gottschalk, 2013). Furthermore, for the harvest clarification, single-use depth filtration or centrifugation devices are frequently used (Liu et al., 2010; Shukla and Thömmes, 2010). Depth filters can absorb the soluble impurities like proteins or DNA. Recently, flocculants are developed and used for primary recovery step (Liu et al., 2010), however, removal and analysis of these flocculants add an additional analytical step.

Due to the employment of innovative technologies starting from cell line engineering to USP development approaches allows an enhanced mAb production from 50 mg/L (in 1986) to 5–20 g/L today (Wurm, 2004; Kelley, 2009; Chon and Zarbis-Papastoitsis, 2011). However, maintaining product quality and impurity level due to very high expression remains challenging.

## Purification Process Development

The next significant step in the product development cycle is the downstream process development, which mainly focuses on the process and product related impurity removal and developing a high yielding with the least impurity purification process. Various innovative approaches are implemented for an efficient and economical downstream process development. This includes developing a platform process and QbD and DOE based high throughput experimental design in a purification process. In addition, single-use system, integration of modeling and replication of mini-plant or pilot plant facilities are applied in downstream processing (del Val et al., 2010; Shukla and Thömmes, 2010; Bhambure et al., 2011). Conventionally, a mAb or any other proteins, including recombinant enzymes are purified using various chromatography, filtration as well as membrane-based purification steps. Additional steps, such as virus inactivation by virus filters (commercially available) and diafiltartion for the final filtration are used for mammalian based product purification as these proteins are expressed from animal cells (Sommerfeld and Strube, 2005; Birch and Racher, 2006; Jain and Kumar, 2008; Gottschalk, 2009; Liu et al., 2010; Shukla and Thömmes, 2010; Chon and Zarbis-Papastoitsis, 2011). However, virus filtration is not required for those proteins produced from bacterial and yeast expression systems.

### Harvest Clarification

The downstream purification process in biopharmaceutical production mainly includes two major steps, chromatography and filtration. Filtration includes, harvest clarification for biomass removal, virus filtration to remove the viruses and tangential flow filtration for protein concentration and polishing step. The centrifugation, TFF-MF, and depth filtrations are the common techniques used for primary cell clarification. While in most of the cases depth filtration and bio-burden reduction filters help in secondary clarification process (Pegel et al., 2011; Tomic et al., 2015; Dhanasekharan et al., 2016).

The TFF-MF separates the particles based on the size exclusion concept. This process utilizes micro filtration membrane having a pore size of up to 0.65 μm. The process is highly efficient and removes whole cell mass and its fragments. This device also offers advantages in scaling up the process due to the modularity of the devices, but with a higher cell density ruptured and fragmented cells are observed in recirculation loop which makes secondary clarification as a necessary step to reduce the smaller cell particles before taking it for the sterile filtration. Product recovery might be low due to increased pellet volume and high desludge, which is common for cell harvest with very high solid content (Pegel et al., 2011; Dhanasekharan et al., 2016; Collins and Levison, 2016).

Centrifugation is the first step used for the separation of the cell biomass produced from the fermentation bacterial and yeast broth (Prasad, 2010; Saraswat et al., 2013). Continuous centrifugation is used for the cell removal in pilot or large scale production processes. Generally three types of centrifugation device, tubular bowl, disk stack, and basket centrifuges are commonly used for the cell clarification or biomass recovery. Each of them used based on the culture type and applications. Recently, a single use continuous centrifugation device is developed by KSep which can be used from low to high speed for various applications. This is a fully automated system and designed to recover over 97% of the product/cell biomass. This system is used for the harvest clarification of recombinant proteins and vaccines^4^.

In the majority of the cases, depth filtration is added after centrifugation step for the loading of clearer materials. Depth filters are in general made up of cellulose, such as diatomaceous earth a porous filter aid and an ionic charged resin binder. Now-a-days depth filtration is widely used as a single use source for the cell clarification in the manufacturing of biopharma drugs and other recombinant proteins. These filters can be used directly with the entire cell broth generated from the fermentation to get cleaner output. Furthermore, primary depth filters are used to remove the bigger particles followed by a secondary depth filter to remove the fine suspended particles. With the advancement of single-use technology, primary and secondary filters are merged into one single step which allows reduction of cycle time and required filter area for an efficient cell clarification and biomass separation. Furthermore, less volume of buffer required for flushing in the process, contributes to the economical clarification of high cell density cultures. The depth filters offers various advantages such as consistent performance, easy scale-up, high product quality and high recovery, smaller footprint in the manufacturing and economical process development which ultimately transformed into low cost and affordable protein production (Dhanasekharan et al., 2016; Collins and Levison, 2016).

Recombinant proteins, including industrial enzymes produced by *E. coli* resulted in the accumulation in the intracellular compartment in the form of inclusion bodies. The protein expressed intracellular requires cell disruption and lysis in order to recover and isolate the inclusion bodies. Numerous cell disruption techniques are being used for the cell lysis followed by protein purification (Shuler and Kargı, 2002; Prasad, 2010).

The recombinant protein expressed in *E. coli* is generally accumulated as inclusion bodies. Since these inclusion bodies are biologically inactive, therefore, *in vitro* refolding of these proteins are required to make it biologically active (Fahnert et al., 2004; Singh and Panda, 2005; Ledung et al., 2009; Singh et al., 2015).

To make the protein biologically active, the solubilized proteins are refolded by removing the chaotropic agents described above. For the refolding of solubilized proteins, various refolding techniques are frequently employed for the renaturation of denatured proteins (Basu et al., 2011; Rathore et al., 2013).

### Chromatographic Separations

Affinity chromatography is a simple and the first step of purification which is used for the capture of a wide variety of recombinant proteins, this step enables purification of recombinant protein with elevated purity in one step. A chromatographic separation resin is used for the capture of expressed protein available after harvest clarification from the upstream bioreactor (Sommerfeld and Strube, 2005; Shukla et al., 2007; Shukla and Hinckley, 2008; Kelley, 2009; Liu et al., 2010; Shukla and Thömmes, 2010). A most popular Protein-A capture step is used for mAb capture from the clarified cell culture harvest. Similarly, other recombinant proteins are also captured using affinity chromatography. Various resin chemistry now-a-days commercially available from different sources which are to be screened and evaluated critically before freezing the first step of the chromatographic operation. The dynamic binding capacity (DBC), yield, quality, host cell protein (HCP) removal and purity are to be used for the resin selection. In addition, most importantly Protein-A leachability are to be tested for every resin and before selection based on the above criteria. The extent of protein aggregate formation should also be one of the important criteria for Protein-A resin selection. The DBC of these resins ranges from 15 to 100 g mAb/L resin depending on type of the mAb, adsorbent, and flow rate (Lain et al., 2009; Liu et al., 2010; Royce, 2014). The yield of the desired product is consistently higher than 95%. Process related impurities such as host DNA, HCPs, virus particles and medium components are removed during Protein-A purification step (Shukla and Hinckley, 2008; Liu et al., 2010; Tarrant et al., 2012).

The major drawback of the Protein-A resin is leachable and non-specific binding host DNA and HCP unwanted impurities, which reduces resin’s DBC and required to be removed in consecutive purification steps (Ghose et al., 2006; Liu et al., 2010; Tarrant et al., 2012). Recently, single use column chromatography is also developed and being used for Protein-A chromatography.

Further, the impurities and unwanted product isoforms are removed by another step Cation exchange chromatography (CEX) which also represents a substitute to Protein-A chromatography (Lain et al., 2009; Liu et al., 2010; Chon and Zarbis-Papastoitsis, 2011; Lain, 2013). Screening and optimizations of the resin can handle up to 100 g/L at high flow rates and purity (Jackewitz, 2008; Lain et al., 2009; Gagnon, 2010; Liu et al., 2010; Lain, 2013). The CEX chromatography is used for the separation of mAb charge variants or aggregates. The percentage of charge variants can be increased or decreased by using pooling strategies and thus desired ratio of these variants are purified after process optimization. This technique is best suited for removal of the mainly negatively charged impurities present in the product (Liu et al., 2010). The CEX resins available in the market are relatively much cheaper than Protein-A resin (Chon and Zarbis-Papastoitsis, 2011). For cost-effective process development, instead of Protein-A chromatography, CEX chromatography can be used as a protein capture step. So far two commercially available mAbs Synagis and Humira are purified by using CEX chromatography as a capture step (Liu et al., 2010; Chon and Zarbis-Papastoitsis, 2011). Subsequently, an ion exchange chromatography (IEC) is frequently used for the removal of residual impurities such as product related impurities, remaining HCP and host DNA, leached Protein-A, media components, endotoxins, and viruses present in the CEX purified protein samples (Ahamed et al., 2007; Liu et al., 2010). Cation or anion exchange chromatography can be used either in bind-and-elute or flow through mode. Flow through elution mode is used in most of the anion exchange purification processes, which removes remaining impurities and gives more than 95% recovery. Another chromatography such as HIC is also used as polishing step for removal of aggregates and product-related impurities. This chromatography is relatively less expensive than Protein-A chromatography (Liu et al., 2010). Downstream optimization includes, screening and selection of appropriate resins, selection of improved ligands and a suitable purification condition which can give optimum yield without much loss of the desired product. The above approach may help to develop the process with shorter residence time, longer lifecycle and high flow rates (Hober et al., 2007; Low et al., 2014). In addition, the attention should be directed towards increasing the resin binding capacity, a number of cycles and establishing an intermediate washing step for the removal of both products as well as process related impurities (Liu et al., 2010; Tarrant et al., 2012).

Another polishing step chromatography called HIC is used for the purification of recombinant proteins based on relative hydrophobicity of the molecules. HIC is used for both small and large scale purification, including hormones, and industrial enzymes (Roettger and Ladisch, 1989; Bhuvanesh et al., 2010). Several recombinant proteins such as anthrax protective antigen, human interferon alpha, etc. have been expressed in *E. coli* were purified employing HIC (Gwinn et al., 2006; Salunkhe et al., 2009; Bhuvanesh et al., 2010; Wang et al., 2014).

The size exclusion or gel filtration chromatography (SEC) separates recombinant protein depend on the molecular weight of the recombinant proteins (Wang et al., 2008). In this chromatography step, the large size proteins are expelled from the resin, where as intermediate size protein can partly enter to the resin and only small size protein can freely enter to the matrix of the resin. This chromatography step is used for the purification of numerous protein, including, single chain variable fragment (ScFv), insulin like growth factor receptor produced from *E. coli* (Levin et al., 2015).

### Membrane Chromatography

Recently, membrane-based chromatography purification steps are developed for a cost effective and speedy purification process (Fröhlich et al., 2012; Gagnon, 2012; Low et al., 2014), this trend can reduce or even eliminate the resin based column chromatographic operations. The membrane chromatography is proven to handle higher feed volumes; therefore, this option is used to handle a greater purification volume with the higher product titers. This approach may lead to cost reduction thus an economical purification process development.

A specific ligand is attached to the convective membrane pores of the symmetric microfiltration membranes (Liu et al., 2010; Cramer and Holstein, 2011; Drioli et al., 2011; Fröhlich et al., 2012). To remove the contaminants, membrane absorbers are used as polishing step (Cramer and Holstein, 2011; Fröhlich et al., 2012). The process and product-related impurities such as viruses, endotoxins, host DNA, HCP, and leached Protein-A binds to the membrane at neutral to slightly basic pH and low conductivity. In the membrane chromatography methods, flow distribution, membrane size distribution and thickness need to be optimized for efficient purification of products (Liu et al., 2010).

The membrane is also used in the various steps of the product development cycle, for example, microfiltration membranes are used for media and buffer filtration in USP. In the downstream process they are used for harvest clarification to remove the cell biomass followed by media particles and DNA before chromatographic step. Further, in subsequent purification steps, ultrafiltration membranes are used for the concentration and diafiltration of the recombinant products (Liu et al., 2010; Cramer and Holstein, 2011; Fröhlich et al., 2012). The other membranes commonly used in purification process are depth filters or high-performance tangential flow membranes (Liu et al., 2010; Cramer and Holstein, 2011; Schreffler et al., 2015).

## Process Development Using Single Use Systems

Increasing demand for new biologics and biosimilars for the mAbs and other recombinant proteins including enzymes have put a tremendous pressure to the industries to manufacture low-cost affordable proteins. With the use of single-use technologies and continuous upstream processing it has become easy to reduce the production cost significantly. However, the adoption of such devices in the purification process with minimal, partly due to concern associated with cost and scale-up challenges (Jacquemart et al., 2016). To address such issues in upstream and downstream processes various innovative approaches are implemented which is described in the subsequent paragraph.

### Single-Use in Upstream Processes

Traditionally, the upstream manufacturing capability is increased by using larger volume bioreactor vessels to meet the market demand for biopharmaceutical drugs. For instance, 10000–25000 L stainless steel bioreactors are used for 7–21 days with the yield of 2–6 g/L for commercial production of mAbs (Rose et al., 2003; Shimoni et al., 2013). With the application of upstream perfusion process together with the advancement of the development of a high producer cell line and medium feed development the upstream productivity per volume increased significantly, which reduces the high volume unit requirement (Low et al., 2014). In perfusion culture, the new media is supplied to the bioreactor in continuous mode which enables increased cell density up to 10–30 times as compared to the conventional batch and fed-batch processes (Lim et al., 2011). The perfusion process allows sustainable cell culture process, which gives up to 4-fold higher productivity due to increased cell density as compared to fed batch process with the same reactor volume (**Figure 3**) (Rose et al., 2003), thus the same quantity can be produced with smaller footprint and low capital cost without compromising with the product quality (**Table 3**). The perfusion process also makes developing easy continuous manufacturing processes. Several products are commercialized by large biopharma companies such as Pfizer, Genentech, Shire, and Genzyme/Sanofi (Bonham-Carter and Shevitz, 2011; Levine et al., 2012; Warikoo et al., 2012) as well as small companies and innovative vaccine manufacturers such as CMC Biologics and Crucell (Langer and Rader, 2014; Pralong et al., 2014). However, still there are several drawbacks to the technology, for example, handling large volumes of medium and purification development, also a high level of operator training is required due to the complexity of the processes (Rose et al., 2003; Nema et al., 2007).

### Single-Use in Downstream Processes

In the purification process, harvest clarification, protein capture, and polishing steps can be optimized by using high throughput, single-use and continuous technologies (**Table 3**). For clarification, filtration is an alternative device to the conventional centrifugation due to ease of handling and single use continuous processing. The single-use disposable filtration systems offer more flexibility and scalability of the clarification process. Recently developed single use continuous centrifugation device developed by KSep (**Figure 3**) can also be used for an efficient harvest clarification and good recovery of the recombinant proteins and vaccines produced for various applications. Some of the technologies such as, the Stax disposable depth filter system (Pall) is a versatile, robust platform that can be operated in different modes depending on the process (Muhl and Sievers, 2010). The Millipore’s Clarisolve as well as D0HC and X0HC adsorptive depth filters can be also used for primary or secondary clarification. These filters allow efficient cell clarification by reducing the cell biomass, HCP and host DNA, and removes most of the cell debris to enable easy load in the chromatographic column (Schreffler et al., 2015). Moreover, filter aids like diatomaceous earth is added to the cell culture, harvest which prevent blockages of the depth filters, therefore allowing easy clarification of the cell culture, harvest with the maximum efficiency in single use formats as demonstrated by Sartoclear Dynamics (Sartorius Stedim Biotech) (Minow et al., 2014; Jacquemart et al., 2016). Each of the technology has their own advantages and disadvantages, therefore it is recommended to evaluate each of the above devices and select any one which is suitable for a particular process and cell type. The performance of the above single-solution depends on the USP performance, cell density, viability and the extent of the cell debris present in the fermentation or bioreactor broth.

Recently developed, simulated moving bed (SMB) technology offers a fully continuous purification process. The BioSMB supplied by Pall life allows continuous loading as well as elution as multiple Protein-A columns are cycled through the standard load, wash, and elution stages at different times (Klutz et al., 2015). The Accelerated Seamless Antibody Purification (ASAP) process is an entirely single-use continuous mAb downstream process, based on AKTA periodic counter-current chromatography (PCC), including Protein-A, mixed mode, and anion exchange resin columns where the three columns are cycled simultaneously (Mothes et al., 2016). Another advantage of the continuous mode is that purification columns are connected in series which allows the use of entire capacity of the resins in each column (Angarita et al., 2015) thus shorter bed heights and cost effective DSP development. The continuous chromatography is advantageous compared to batch resin chromatography, as SMB resulted 30% high productivity, up to 40% increased loading capacity, and up to 27% less buffer consumption (Angarita et al., 2015; Kaltenbrunner et al., 2016).

The integration of innovative single-use approach in upstream and downstream processing provides an opportunity to develop a flexible and small foot print facility which ultimately is advantageous in manufacturing cost-effective and affordable drugs (Zhao et al., 2015). To enable producing more affordable drugs in the existing conventional facility, advanced single-use technologies can be incorporated and can smoothly transform into a more economical processing (**Table 3**). Recently, an analysis done by Biosolve shows that the operating cost of per gram of mAb for a single-use facility is 22% lower as compared to a stainless steel facility due to less work horse, utility requirement, maintenance, and waste generation (Levine, 2013).

## Continuous Manufacturing

Now-a-days there is much talk about continuous manufacturing in the area of biopharmaceutical development, since continuous manufacturing can help reduce the manufacturing footprint, lower capital and operating costs, enhanced product quality, better scalability and make possible time to the market.

The continuous manufacturing is used by many companies as an alternate process for the batch and fed-batch processes for the economical biopharmaceutical development. The upstream perfusion process is a quite old technology, which has been used for over two decades for various product development, however, the development of the continuous purification process (**Figure 3**) is relatively an innovative approach which is used in combination with the perfusion based cell culture technology. In a continuous cell culture process, the raw materials such as medium and feeds are continuously fed into a cell culture vessel while expressed protein is removed continuously on the other side. The continuous cell culture process is run for over a month or so depending upon the cell line sustainability and the optimized process which can give a large volume of the harvest containing desired quantity of the proteins. The continuous USP is also advantageous in terms of producing better quality product as the waste material is removed from the vessel continuously which may hamper the product quality in a batch or fed-batch processes. Also in this process the cell density increases several folds as compared to batch and fed-batch processes which in turns beneficial in increasing the volumetric productivity of the desired proteins.

Furthermore, the continuous upstream perfusion based cell culture process is integrated to the downstream processing in a continuous mode, which includes, harvest clarification and filtration and column chromatography for product purification. Although, many hurdles are faced during the process optimization, but due to innovation in the purification devices and single use system, it has become easy to integrate upstream and downstream processes in a continuous bioprocessing mode. Various new purification devices such as PCC (by GE healthcare), Inline conditioning system (ILC, by GE), BioSMB (by Pall life science) are developed especially for the continuous bioprocessing of the upstream materials (Klutz et al., 2015). The chromatography systems are inbuilt with the 4–8 columns and software for ease of operation and purification in a continuous mode.

The continuous manufacturing offers several advantages over batch and fed-batch processes. For example, the size of continuous systems is comparatively much smaller than batch systems, consequently they can be used for the production of a large or small amount depends on the requirement (Jin et al., 2010). In addition, the smaller vessel size demands lesser complex setup cycles, thus requires limited scale-up from clinical manufacturing, which allows speedy development and launch in the market and could cater the high market demand (Tscheliessnig et al., 2013), however, the technology demands much greater time and initial investment for the process development.

Also, the footprint of the continuous manufacturing facility can be reduced as low as up to 40–90% and capital expenditures estimated to be 20–76% lowers than for batch systems (**Table 3**) (Birch and Racher, 2006; Shukla et al., 2007). The continuous manufacturing requires more time in batch processing (30–60 days) compared to batch and fed batch processes (10–18 days) as this runs for several weeks in continuous mode with high cell viability. For example, a 5 L bioreactor can produce 5000 L harvest in a continuous mode. In spite of various advantages, continuous manufacturing, however, has its own disadvantages and challenges such as (1) Quality and regulatory challenges when switching from batch to continuous process in a production facility. (2) Defining the batch for quality control when a product is recalled from the market. (3) It may not be feasible for low volume and high-value products as change over and loss in product during start may have potential value and (4) it requires a holistic and integrated multi-disciplinary approach across engineering, technical and manufacturing disciplines. The US-FDA regulatory bodies encourage the biological industries for the production of new products using continuous manufacturing approach. In addition, the FDA also recommends using QbD approach for a consistent, continuous manufacturing as a more innovative manufacturing approach to improve the assurance of consistency and quality of the product.

## Conclusion

Industrial production of recombinant products including mAbs are speedily growing in both upstream and downstream processings. Availability of various expression systems (*E. coli, S. cerevisiae, P. pastoris*, and CHO) enables selecting an appropriate host for high level of protein expression. Gene editing and cell line engineering of these hosts can potentially improve the product yield and quality which may allow easy upstream and downstream processes development. Various high throughput devices such as Biolector, AMBR, and AKTA systems are now-a-days available for an efficient upstream and downstream process development. These innovative approaches can be used for a successful and economical drug development. Single-use technologies are progressively more adopted in both upstream and downstream operations, which increases the flexibility and speed while reducing capital cost and down time. The innovative continuous processing is also adopted by several biopharma companies which can be beneficial in reducing the manufacturing footprint, capital as well as labor cost. The commercial launch of the new perfusion devices, and continuous chromatographic system such as PCC and BioSMB have made the downstream processing easier for a continuous operation. The QbD approach is highly recommended by regulators for a consistent process and good quality product development. The biopharmaceutical industries continue to shift towards more flexible, automated platforms and economical product development, which in turn can help in developing the cost effective processes and affordable drug development for a large community.

## Author Contributions

All authors listed have made a substantial, direct and intellectual contribution to the work, and approved it for publication.

## Conflict of Interest Statement

The authors declare that the research was conducted in the absence of any commercial or financial relationships that could be construed as a potential conflict of interest.

## Footnotes

1 https://www.thermofisher.com/in/en/home/life-science/protein-biology/protein-expression/yeast-protein-expression.html

2 http://www.m2p-labs.com/bioreactors/products/biolector/

3 https://www.thermofisher.com/in/en/home/life-science/protein-biology/protein-expression/yeast-protein-expression/pichiapink-yeast-expression-systems.html

4 www.sartorius.com
